# Small Molecule B-RAF Inhibitors as Anti-Cancer Therapeutics: Advances in Discovery, Development, and Mechanistic Insights

**DOI:** 10.3390/ijms26062676

**Published:** 2025-03-16

**Authors:** Yamile Abuchard Anaya, Ricardo Pequeno Bracho, Subhash C. Chauhan, Manish K. Tripathi, Debasish Bandyopadhyay

**Affiliations:** 1 School of Integrative Biological and Chemical Sciences, The University of Texas Rio Grande Valley, 1201 West University Drive, Edinburg, TX 78539, USA; yamile.abuchardanaya01@utrgv.edu (Y.A.A.); ricardo.pequenobracho01@utrgv.edu (R.P.B.); 2 Department of Health and Human Performance, College of Health Professions, The University of Texas Rio Grande Valley, 1201 West University Drive, Edinburg, TX 78539, USA; 3 South Texas Center of Excellence in Cancer Research, McAllen, TX 78504, USA; subhash.chauhan@utrgv.edu (S.C.C.); manish.tripathi@utrgv.edu (M.K.T.); 4 Division of Cancer Immunology and Microbiology, Medicine, and Oncology ISU, School of Medicine, The University of Texas Rio Grande Valley, 5300 N L St., McAllen, TX 78504, USA; 5 School of Earth Environment & Marine Sciences, The University of Texas Rio Grande Valley, 1201 West University Drive, Edinburg, TX 78539, USA

**Keywords:** B-RAF, MAPK pathway, B-RAF V600E mutation, RAF kinase inhibitors, small molecule inhibitors, Vemurafenib, Dabrafenib, Encorafenib, cancer therapy, MEK inhibitors, combination therapy, drug resistance, colorectal cancer, melanoma, thyroid carcinoma, non-small cell lung carcinoma, oncology, clinical trials

## Abstract

B-RAF is a serine/threonine kinase that plays a crucial role in the MAPK signaling pathway, regulating cell proliferation and survival. Mutations in B-RAF, particularly V600E, are associated with several malignancies, including melanoma, colorectal cancer, and non-small cell lung cancer, making it a key therapeutic target. The development of B-RAF inhibitors, such as Vemurafenib, Dabrafenib, and second-generation inhibitors like Encorafenib, has led to significant advancements in targeted cancer therapy. However, acquired resistance, driven by MAPK pathway reactivation, RAF dimerization, and alternative signaling pathways, remains a major challenge. This review explores the molecular mechanisms of B-RAF inhibitors, their therapeutic efficacy, and resistance mechanisms, emphasizing the importance of combination strategies to enhance treatment outcomes. The current standard of care involves B-RAF and MEK inhibitors, with additional therapies such as EGFR inhibitors and immune checkpoint blockades showing potential in overcoming resistance. Emerging pan-RAF and brain-penetrant inhibitors offer new opportunities for treating refractory cancers, while precision medicine approaches, including genomic profiling and liquid biopsies, are shaping the future of B-RAF-targeted therapy.

## 1. Introduction

The human protein kinase family, with its 518 genes, plays a crucial role in cell signaling and regulation, making it one of the largest gene families [1]. These kinases are classified into three major groups: tyrosine-kinase-like proteins (43 members), protein-tyrosine kinases (90 members), and protein-serine/threonine kinases (385 members) [1]. Among these, RAF proteins are cytosolic serine/threonine kinases that regulate critical cellular processes. The RAF family consists of three isoforms, A-RAF, B-RAF, and C-RAF, which were first identified in 1983 in studies on retroviral oncogenes [2].

B-RAF is a key component of the mitogen-activated protein kinase (MAPK) signaling cascade, functioning downstream of RAS to regulate cell proliferation, differentiation, and survival [3]. The RAF-MEK-ERK signaling axis is crucial in both normal physiology and tumorigenesis [4]. B-RAF has also been implicated in other signaling pathways, including the Jak/STAT and Wnt/β-catenin pathways, highlighting its role as a central regulatory kinase in various cellular contexts.

The BRAF gene encodes a kinase involved in transmitting extracellular signals to the nucleus, modulating gene expression to regulate cellular functions. Initially, RAF genes were discovered as oncogenes in retroviruses capable of inducing tumors in mice and chickens [5]. The v-RAF gene, identified in the transforming retrovirus Mouse Sarcoma Virus (MSV) 3611, was the first member of this kinase family associated with tumorigenesis [5]. Despite early studies not identifying clear oncogenic mutations in human malignancies, subsequent research established RAF proteins as critical downstream effectors of oncogenic RAS, which is frequently mutated in cancer [5]. This breakthrough led to the identification of activating mutations in B-RAF in approximately 15% of colorectal cancers and 70% of malignant melanomas [5].

B-RAF mutations, particularly the V600E substitution (where valine is replaced by glutamic acid), are the most common and result in constitutive activation of the kinase, driving uncontrolled cell proliferation [6]. Other less frequent mutations include V600K, V600R, V600E2, and V600D, each with distinct biochemical and structural consequences. V600E mutations account for over 90% of all B-RAF mutations, whereas V600K mutations represent 5–30% of cases [6]. These alterations lead to persistent activation of MEK/ERK signaling, promoting tumorigenesis and therapeutic resistance. Understanding the structural and functional impact of these mutations remains an area of active investigation.

Murine models have provided key insights into the oncogenic role of B-RAF mutations. The B-RAF^V600E^ mutation drives an initial burst of cell proliferation via activation of the MEK/ERK cascade and upregulation of cyclin D1, initiating tumorigenesis [7]. However, oncogene-induced senescence (OIS) serves as a barrier to continued tumor growth in early stages [7]. To bypass this, cancer cells must acquire additional genetic alterations, such as the inactivation of tumor suppressors like p16^INK4a^ or p19^ARF^, allowing for malignant progression [7].

Because oncogenic B-RAF is essential for cancer cell survival, it is considered a driver oncogene, making it an attractive therapeutic target [8]. Small molecule inhibitors targeting B-RAF, such as Vemurafenib and Dabrafenib, have revolutionized the treatment of B-RAF-mutant cancers. However, resistance mechanisms often arise through the reactivation of MAPK signaling or alternative survival pathways [9]. Combination therapies involving B-RAF and MEK inhibitors have shown promise in overcoming resistance, paving the way for more effective treatment strategies.

This review discusses the discovery and development of small molecule B-RAF inhibitors, their mechanisms of action, and clinical applications. Additionally, emerging combination therapies and novel strategies to counteract resistance will be explored. Understanding the mechanistic underpinnings of B-RAF inhibition is crucial for advancing targeted therapies and improving patient outcomes.

## 2. B-RAF Mutations in Cancer

RAF kinases, particularly B-RAF, are among the most frequently mutated kinases in cancer [10]. According to The Cancer Genome Atlas (TCGA), the prevalence of oncogenic B-RAF mutations varies significantly across different cancer types. Hairy cell leukemia exhibits the highest frequency, approaching 100%, followed by papillary thyroid cancer (40–45%), cutaneous melanoma (~50%), and Langerhans cell histiocytosis (39%) [10,11,12]. While less common, B-RAF mutations remain clinically significant in colorectal cancer (5–15%) and non-small cell lung cancer (1–3%) [10,11,12,13]. These mutations often serve as key oncogenic drivers, with varying degrees of sensitivity to targeted therapies. Additionally, B-RAF mutations are implicated in developmental disorders such as cardio-Facio-cutaneous syndrome, Noonan-like syndrome (NLS), and LEOPARD syndrome [14].

In normal cells, B-RAF plays a pivotal role in regulating cell growth by modulating the mitogen-activated protein kinase (MAPK) signaling pathway [8]. However, oncogenic mutations, particularly V600E, lead to constitutive activation of this cascade, resulting in uncontrolled proliferation, enhanced invasion, metastasis, and apoptosis resistance [8]. Given its central role in tumorigenesis, B-RAF has emerged as a crucial therapeutic target, with small molecule inhibitors demonstrating efficacy in various malignancies [8]. However, treatment resistance remains a challenge, necessitating combination strategies with MEK inhibitors or alternative targeting approaches to improve therapeutic responses [8].

The most well-characterized B-RAF mutation is V600E, which is highly oncogenic and found in multiple cancers. Other variants, such as V600K, occur in melanoma (~5–30%) and are also targetable with B-RAF and MEK inhibitors. Non-V600 mutations are found across multiple cancer types, including lung, colorectal, thyroid, and melanoma, often exhibiting weaker kinase activity and varying responses to therapy. Additionally, rarer mutations such as F595L, L597Q, and G469A occur in melanoma and colorectal cancer but show reduced sensitivity to B-RAF inhibitors [11,12,15]. Beyond B-RAF, C-RAF and A-RAF mutations are rare but can be found in lung, ovarian, pancreatic, and glioma cancers, often presenting challenges due to RAF dimerization and limited therapeutic options [16,17]. The summarized details of B-RAF and related mutations are presented in Table 1, which serves as a visual reference for their clinical significance and prevalence across different cancers.

### 2.1. Melanoma

Melanoma is the cancer most driven by B-RAF mutations, with an incidence exceeding 50% [19]. The V600E mutation, accounting for ~90% of B-RAF alterations in melanoma, leads to constitutive activation of the MAPK signaling pathway [19]. This mutation contributes to disease progression by promoting immune evasion, sustained MEK/ERK pathway activation, angiogenesis via HIF-1α and VEGF upregulation, enhanced migration, and resistance to senescence and apoptosis [19]. Notably, B-RAF mutations are rare in melanomas arising from acral and mucosal sites but more prevalent in tumors developing on intermittently sun-exposed skin [19]. Additionally, the V600K mutation occurs in 5–30% of melanoma cases and is also targetable with B-RAF and MEK inhibitors [11,12].

### 2.2. Hepatocellular Carcinoma

While B-RAF mutations are rare in hepatocellular carcinoma (HCC), hyperactivation of the RAS/RAF/MEK/ERK pathway is frequently observed [20]. This pathway, often influenced by aberrant C-MET signaling, drives angiogenesis, anti-apoptotic mechanisms, and cancer cell proliferation [20]. A TCGA study of 363 HCC samples found a low B-RAF mutation prevalence of 0.3%, indicating that while B-RAF mutations are not a primary driver of HCC, they may still contribute to tumor biology in select cases [20].

### 2.3. Colorectal Cancer

Colorectal cancer (CRC), a leading cause of cancer-related mortality in young adults, harbors B-RAF mutations in approximately 5–15% of metastatic cases [21]. The vast majority (90%) of these mutations involve V600E, which is associated with poor prognosis and resistance to standard therapies [21]. B-RAF-mutated CRCs exhibit distinct clinical and pathological features, disproportionately affecting elderly women, individuals of Caucasian descent, and right-sided primary tumors [21]. Unlike other CRC subtypes that predominantly metastasize to the liver, B-RAF V600E-mutated CRCs have a higher propensity for peritoneal dissemination [21]. Non-V600 mutations, which occur in 5–20% of cases, may exhibit weaker kinase activity and sometimes respond better to MEK inhibitors than B-RAF inhibitors [11,12].

### 2.4. Thyroid Cancer

Thyroid carcinoma, the most common endocrine malignancy, frequently harbors B-RAF mutations, with reported prevalence ranging from 40% to 45% [22]. The T1799A transversion, leading to the V600E substitution, is the most prevalent genetic alteration in papillary thyroid carcinoma (PTC) [22]. B-RAF mutations drive thyroid tumorigenesis through persistent MAPK pathway activation, underscoring the potential of targeted therapies aimed at disrupting this oncogenic signaling axis [22].

## 3. Challenges in Targeting B-RAF

The initial rationale for targeting B-RAF in cancer therapy was based on the premise that inhibiting its activity would downregulate the RAS/RAF/MEK/ERK signaling cascade, thereby suppressing tumor progression. However, resistance to B-RAF inhibitors has emerged as a major challenge, limiting their long-term efficacy. Multiple resistance mechanisms, including secondary mutations, the activation of bypass signaling pathways, and tumor microenvironment adaptations, enable cancer cells to evade B-RAF inhibition and sustain oncogenic signaling [23,24].

### 3.1. Mechanisms of B-RAF Inhibitor Resistance

One of the primary resistance mechanisms involves secondary mutations that sustain reliance on the MAPK pathway. Acquired mutations in NRAS or MAP2K (MEK1/2) restore downstream signaling despite B-RAF inhibition [24]. Among the most common resistance-associated genetic alterations, mutually exclusive mutations in RAS (25%) and B-RAF V600 (22%) were identified, leading to continued activation of the MAPK cascade [24].

Beyond genetic mutations, alternative signaling pathways can compensate for B-RAF inhibition. Notably, the Wnt/β-catenin and JAK/STAT pathways contribute to drug resistance, enabling tumor cells to escape dependence on the MAPK pathway [23]. Recent studies have shown that treatment with B-RAF inhibitors, including both first-generation and next-generation compounds, leads to the activation of focal adhesion kinase (FAK), which in turn upregulates the Wnt/β-catenin pathway in B-RAF V600E-mutant colorectal cancer (CRC) cell lines [23]. Additionally, the activation of the PI3K/Akt signaling pathway was implicated in resistance to both B-RAF inhibitors and B-RAF/MEK inhibitor combinations, further underscoring the complexity of resistance mechanisms [24].

### 3.2. Tumor Microenvironment and Adaptive Resistance

The tumor microenvironment plays a crucial role in adaptive resistance to B-RAF inhibitors. In metastatic melanoma, drug-resistant tumor cells exhibit increased secretion of CCL2 chemokine, which promotes an immunosuppressive environment that enhances drug resistance [25]. Furthermore, PD-L1 upregulation in melanoma cells leads to T-cell exhaustion, reducing the immune system’s ability to eliminate resistant cancer cells [25]. Another contributing factor is the presence of tumor-associated macrophages (MDSCs), which secrete tumor-promoting cytokines such as nitric oxide (NO) and interleukin-10 (IL-10), accelerating melanoma progression and reinforcing resistance [25].

One of the most significant findings in resistance mechanisms is the role of epidermal growth factor receptor (EGFR) overexpression. A study analyzing biopsies from 16 patients with B-RAF-mutant melanoma treated with either Trametinib (a MEK inhibitor) or Dabrafenib/Vemurafenib (B-RAF inhibitors) found that six post-treatment samples exhibited a significant increase in EGFR expression, as determined by immunohistochemistry (IHC) [26]. This suggests that tumors may evade B-RAF inhibition by upregulating EGFR, leading to sustained MAPK pathway activation and enhanced metastatic potential [26]. Notably, given their impact on immune surveillance, baseline patient variables such as LDH levels and tumor burden may affect how well patients respond to treatment with B-RAF inhibitors or MEK inhibitors [27]. Therefore, because B-RAF-MEK inhibition significantly alters antigen presentation and, subsequently, immune response, it can play a significant role in the tumor microenvironment [27].

### 3.3. Overcoming Resistance: Combination Therapies

Given the rapid emergence of B-RAF inhibitors, ongoing research is exploring combination therapies as a potential solution. One promising strategy involves the co-administration of B-RAF and MEK inhibitors, which has demonstrated improved clinical outcomes by preventing paradoxical MAPK activation [28]. Among these, MEK162, an oral small molecule inhibitor developed by Novartis, has shown efficacy in inhibiting MEK1 and MEK2 and is currently being investigated in combination with LGX818, a selective B-RAF inhibitor, for metastatic melanoma treatment [28].

Overall, understanding the diverse mechanisms of B-RAF inhibitor resistance is critical for developing more effective therapeutic strategies. Future efforts will likely focus on targeting compensatory pathways, refining combination regimens, and leveraging immunotherapeutic approaches to enhance treatment efficacy and overcome resistance [23,24,25,26,28].

## 4. Discovery and Development of B-RAF Inhibitors

The development of B-RAF kinase inhibitors has significantly improved targeted cancer therapies, particularly for malignancies driven by B-RAF V600 mutations. These inhibitors disrupt the RAS/RAF/MEK/ERK signaling cascade, which plays a crucial role in tumor growth and progression. To date, two major categories of B-RAF inhibitors have been developed: type I and type II inhibitors [29].

### 4.1. Classification of B-RAF Inhibitors

Type I B-RAF inhibitors selectively bind to the active (DFG-in) conformation of the kinase, forming hydrogen bonds with the kinase hinge residues and interacting with the ATP-binding site via hydrophobic interactions in and around the adenine ring [29]. These inhibitors exhibit high specificity for the B-RAF kinase and have demonstrated higher response rates compared to type II inhibitors [29].

Conversely, type II B-RAF inhibitors bind to the inactive (DFG-out) conformation, establishing hydrogen bonds with residues in the DFG motif and the C-helix [29]. Through Van der Waals interactions, type II inhibitors interact with an allosteric site adjacent to the ATP-binding pocket, extending into the adenine region and forming additional hydrogen bonds with kinase hinge residues [29]. The DFG-out conformation creates a unique allosteric pocket, which was identified as a “RAF-selective pocket” containing the residues Thr529, Leu514, Phe595, Gly593, and Leu505, offering new potential targets for drug design [30].

### 4.2. Generations of B-RAF Inhibitors

The discovery and refinement of B-RAF inhibitors have evolved through three generations, each improving specificity, overcoming drug resistance, and expanding treatment efficacy.

- First-Generation B-RAF Inhibitors:

Initially, B-RAF inhibitors targeted RAF mutants, including C-RAF, within the MAPK pathway. These ATP-competitive small molecules adopt an αC-IN conformation, binding to active-site protomers within RAF dimers [31]. However, they lacked selectivity for mutant B-RAF monomers, resulting in insufficient suppression of RAF dimers and paradoxical activation of the MAPK pathway [31]. The first B-RAF inhibitor studied in melanoma clinical trials was Sorafenib (Nexavar), a multi-kinase inhibitor targeting RAF, VEGFR, and other receptor tyrosine kinases (RTKs) [28]. Although initially promising, Sorafenib was ultimately less effective in melanoma compared to specific B-RAF inhibitors combined with MEK inhibitors, which became the standard of care [28].

- Second-Generation B-RAF Inhibitors:

The discovery of B-RAF V600E mutations in 2002 led to the development of second-generation inhibitors with increased selectivity for mutant B-RAF [31]. These inhibitors, which include Vemurafenib (PLX4032) and Dabrafenib, utilize an αC-out binding conformation, improving target selectivity for B-RAF V600E-driven tumors while avoiding off-target effects seen with first-generation inhibitors [31]. These inhibitors dramatically improved clinical outcomes in B-RAF V600-driven melanoma and certain solid tumors, particularly when used in combination with MEK inhibitors [31].

- Third-Generation B-RAF Inhibitors:

Designed to overcome paradoxical activation and B-RAF dimerization, third-generation inhibitors primarily adopt an αC-IN/DFG-OUT conformation [17]. These inhibitors are highly selective for RAS-dependent B-RAF dimers and B-RAF monomers, making them effective against tumors resistant to earlier B-RAF inhibitors [17]. Unlike their predecessors, third-generation inhibitors have a narrower therapeutic window, requiring optimized combination therapy strategies [17].

### 4.3. High-Throughput Screening and Preclinical Development

Following the discovery of B-RAF V600 mutations, researchers employed high-throughput kinase screening assays to evaluate 20,000 small molecules (150–350 Daltons) for B-RAF inhibition [32]. Of these, over 280 compounds demonstrated direct interactions with B-RAF during preclinical studies [32].

A critical discovery was that the 7-azaindole group conferred high-affinity binding to the active kinase site of B-RAF [32]. Among the leading candidates, PLX4720 exhibited high selectivity for both wild-type and B-RAF V600-mutant kinases, showing robust inhibition of MAPK pathway signaling in preclinical models [32].

**Figure 1:**
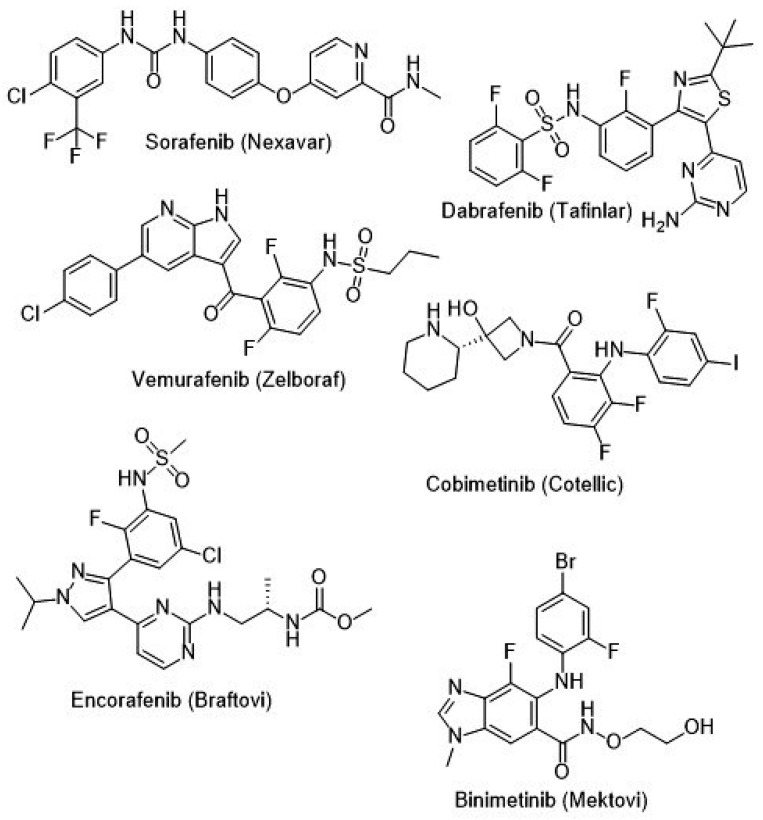
FDA-approved B-RAF inhibitors (December 2024).

The success of PLX4720 led to the development of PLX4032 (Vemurafenib), which was tested in xenograft models and mouse studies [32]. These studies demonstrated that Vemurafenib effectively inhibited melanoma cell proliferation and induced apoptosis by blocking MAPK signaling [32]. Due to compelling preclinical and early clinical trial results, Vemurafenib rapidly received FDA approval for the treatment of B-RAF V600E-mutant melanoma [32]. The B-Raf inhibitors across cancer subtypes are shown in Table 2, whereas Figure 1 shows the latest B-RAF inhibitors approved by the FDA.

## 5. Current Clinical Trials and Combination Therapies

With the emergence of B-RAF inhibitors, significant progress has been made in treating B-RAF-mutant cancers. However, resistance to monotherapy and limited long-term efficacy have necessitated the development of combination strategies. Clinical trials continue to investigate second-generation inhibitors and novel therapeutic combinations, particularly with MEK inhibitors, EGFR inhibitors, immune checkpoint inhibitors, and anti-angiogenic agents.

### 5.1. B-RAF Inhibitors in Clinical Trials

#### 5.1.1. Vemurafenib Clinical Trials (Phase I–III)

Vemurafenib, a selective B-RAF inhibitor, was tested in multiple phases to evaluate its clinical efficacy and safety. Phase I Trial: The first-in-human Phase I trial included 55 patients and was divided into dose-escalation (any tumor genotype) and extension (B-RAF V600-mutant melanoma) cohorts [32]. The results were promising, showing a 69% response rate, with 11 patients experiencing partial responses and one achieving a complete response [32]. The median progression-free survival (PFS) was 7 months, with an 81% response rate in the extension cohort [32].

Phase II Trial: A multi-center trial in 132 patients with previously treated advanced melanoma showed an overall response rate of 53%, including 6% complete response (CR) and 47% partial response (PR). The median PFS was 6.8 months, and 14% of patients experienced disease progression [32].

Phase III Trial: A randomized, multinational trial compared Vemurafenib to dacarbazine, enrolling 680 patients [32]. At 6 months, the survival rate was 84% for Vemurafenib vs. 64% for dacarbazine, leading to FDA approval on 17 August 2011 [32]. In an open-label extension study, 3226 patients with B-RAF V600-mutant melanoma showed a positive therapeutic response, though photosensitivity reactions were a notable side effect [32,41].

#### 5.1.2. Dabrafenib Clinical Trials

Dabrafenib, another ATP-competitive B-RAF inhibitor, was investigated in randomized Phase III trials in stage III-IV melanoma patients [42]. Compared to dacarbazine, Dabrafenib significantly improved PFS (5.1 vs. 2.7 months) [42]. The most common adverse events included fever, fatigue, arthralgia, and squamous cell carcinoma (6%), which were lower than those observed with Vemurafenib (20%) [42].

#### 5.1.3. Encorafenib Clinical Trials (Second-Generation)

Encorafenib (LGX818) is a highly selective, ATP-competitive second-generation B-RAF inhibitor developed after Dabrafenib and Trametinib [43,44]. In the dose-expansion phase (CLGX818x2101 trial), 18 patients with B-RAF-V600E metastatic CRC were treated, resulting in a disease control rate (DCR) of 67% and an overall response rate (ORR) of 5.6% [45]. Encorafenib selectively inhibits B-RAF (IC50 = 0.47 nM), CRAF (IC50 = 0.30 nM), and B-RAF-V600E (IC50 = 0.35 nM) without affecting the RAS/RAF/MEK/ERK pathway in non-B-RAF-V600E-mutant cells [44].

#### 5.1.4. Resistance Mechanisms and Future Directions

While Vemurafenib and Dabrafenib showed unprecedented initial responses, many patients developed acquired resistance due to RAF dimerization, MAPK pathway reactivation, and secondary mutations [43,44]. B-RAF inhibitors must be combined with MEK inhibitors or other targeted therapies to counteract paradoxical activation and resistance [31,46].

### 5.2. Combination Therapy in Clinical Trials

Table 3, Table 4, Table 5 and Table 6 summarize the trial numbers, various drug combinations, and targeting pathways, along with the results of different combination therapies currently under clinical trials.

### 5.3. Phase III Clinical Trials

**Figure 2:**
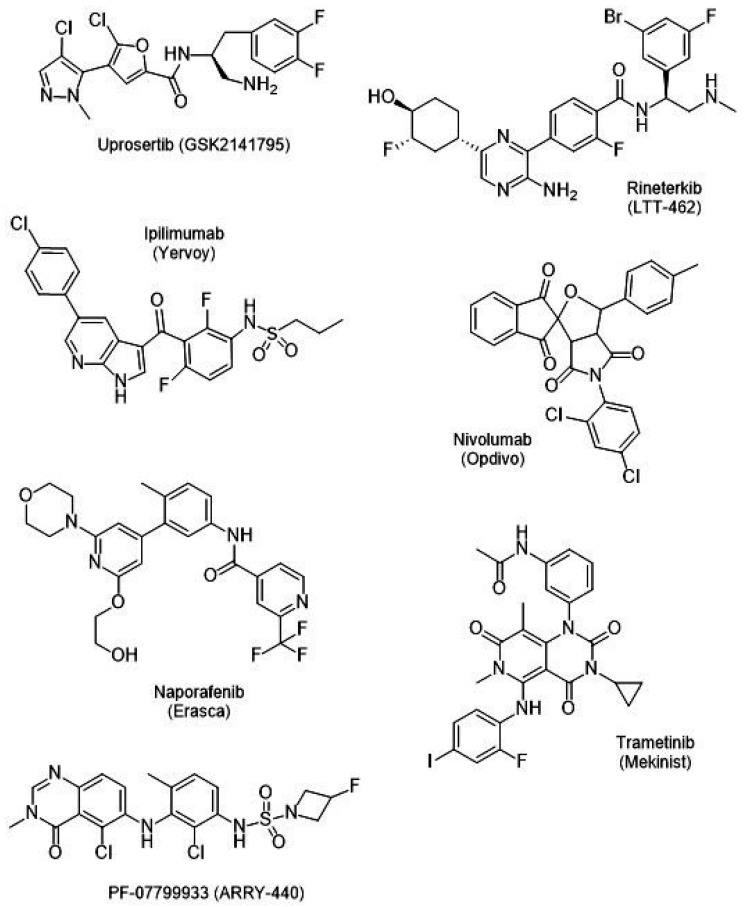
Current clinical–trial potential treatment drugs (Part 1) (December 2024).

**Figure 3:**
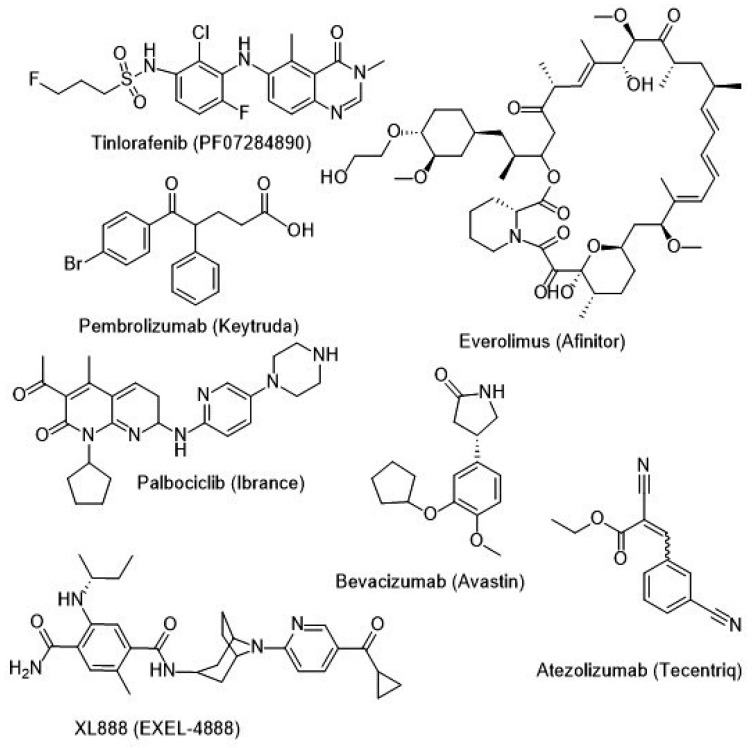
Current clinical–trial potential treatment drugs (Part 2) (December 2024).

Table 7 summarizes the trial numbers, various drug combinations, targeting pathways, and the results of different combination therapies currently under Phase III clinical trials. Figure 2 and Figure 3 show the B-RAF inhibitors in various stages of clinical trials.

## 6. Future Directions and Clinical Implications

Despite the success of B-RAF inhibitors, acquired resistance remains a significant challenge, necessitating next-generation inhibitors and novel combination strategies. Future research should focus on pan-RAF inhibitors that effectively target RAF dimers and prevent MAPK pathway reactivation, which is a key driver of resistance [51]. Additionally, dual and triple therapy regimens incorporating B-RAF inhibitors with MEK inhibitors, immune checkpoint inhibitors (PD-1/PD-L1, CTLA-4), or AKT/mTOR pathway inhibitors have demonstrated promising results in clinical trials and should be further explored [47,49]. Recent Phase II and III trials have highlighted the importance of personalized therapy, where treatment selection is guided by tumor mutational profiling, helping to identify patient subgroups most likely to benefit from targeted interventions [53,54]. Furthermore, emerging brain-penetrant B-RAF inhibitors such as PF-07284890 offer hope for treating B-RAF-mutant brain metastases, a current limitation of existing therapies [55].

The clinical implications of these advances extend beyond melanoma to NSCLC, CRC, thyroid carcinoma, and pediatric gliomas, where B-RAF-targeted therapies are now being actively tested in combination with other targeted agents [36,39,40,52]. In colorectal cancer, for instance, B-RAF inhibitors alone are ineffective due to EGFR reactivation, but the combination of B-RAF + MEK + EGFR inhibitors has demonstrated significant survival benefits, underscoring the necessity of combination regimens in certain tumor types [39]. Additionally, anti-angiogenic therapies such as Bevacizumab have shown potential in enhancing B-RAF inhibitor efficacy, though their clinical role remains to be fully established [56]. The integration of liquid biopsies and circulating tumor DNA (ctDNA) analysis will further allow for real-time monitoring of resistance mechanisms, improving treatment adaptability and precision oncology approaches in B-RAF-mutant cancers [57].

## 7. Conclusions

RAF proteins play a pivotal role in cancer progression, as oncoproteins such as RAS, MYC, and RAF can paradoxically induce apoptosis or senescence when signaling is excessively activated [58]. The RAS/RAF/MEK/ERK (MAPK) signaling cascade is fundamental in regulating cell growth, survival, and differentiation, and dysregulation of this pathway due to B-RAF mutations leads to uncontrolled cell proliferation [59,60]. The B-RAF V600E mutation, commonly observed in thyroid, colorectal, and melanoma malignancies, is particularly aggressive due to its ability to constitutively activate the MAPK pathway [59]. Inhibiting B-RAF is therefore critical, but overcoming resistance mechanisms remains a challenge. The introduction of selective B-RAF inhibitors, such as Vemurafenib and Dabrafenib, has significantly improved treatment outcomes, but acquired resistance limits their long-term efficacy [32,42]. Current research highlights the importance of combination therapies, particularly B-RAF + MEK inhibitors, which remain the standard of care [56]. Additionally, incorporating EGFR inhibitors has emerged as a promising strategy to counteract resistance, particularly in colorectal cancer, where B-RAF inhibition alone leads to feedback activation of EGFR and MAPK signaling [39,61]. Recent breakthroughs in molecular targeted therapy have positioned EGFR inhibitors as one of the most effective clinical tools, underscoring their role in future treatment paradigms [61]. Furthermore, B-RAF inhibition should be studied in the context of multiple interconnected pathways, including MAPK, JAK/STAT, Wnt/β-catenin, and mTOR, to develop more comprehensive treatment approaches [58]. Looking forward, next-generation pan-RAF inhibitors and novel RAF-targeting strategies offer new hope for overcoming resistance. Recent evidence suggests that modern RAF inhibitors may effectively block the RAS pathway, potentially surpassing the efficacy of MEK inhibitors [62]. Additionally, brain-penetrant B-RAF inhibitors such as PF-07284890 are being evaluated in clinical trials, addressing a current limitation in the treatment of B-RAF-mutant brain metastases [55]. As research advances, personalized approaches leveraging tumor genomic profiling, liquid biopsies, and circulating tumor DNA (ctDNA) analysis will optimize treatment selection and adaptability [57]. The future of B-RAF-targeted therapies lies in overcoming resistance through adaptive combination strategies and next-generation drug development, ultimately expanding the potential of precision oncology [52,54].

## Figures and Tables

**Table 1:** Prevalence and clinical relevance of RAF mutations in cancer.

RAF Isoform	Mutation	Cancer Type(s)	Prevalence	Clinical Relevance	References
B-RAF (1983)	V600E	Melanoma	~50%	Highly oncogenic	[1,11,12,15,18]
		Colorectal	~5–15%	Poor prognosis and resistance to B-RAF inhibitor monotherapy	[1,11,12,15,18]
		Thyroid (Papillary)	~40–45%	highly aggressive	[1,11,12,15,18]
		Ovarian	~30%	Sensitive to MEK inhibitors	[1,11,12,15,18]
		Non-small Cell Lung Carcinoma (NSCLC)	~1–3%	Targeted therapy includes B-RAF and MEK inhibitors	[1,11,12,15,18]
B-RAF	V600K	Hairy Cell Leukemia	~99%	Highly sensitive to B-RAF inhibition	[1,11,12,15,18]
B-RAF	Non-V600	Melanoma	~5–30%	Targetable with B-RAF and MEK inhibitors	[1,11,12,15,18]
B-RAF	F595L, L597Q, and G469A	Lung, Colorectal, Thyroid, and Melanoma	~5–20%	Associated with weak/intermediate kinase activity and sometimes respond better to MEK inhibitors instead of B-RAF inhibitors	[1,11,12,15,18]
C-RAF (1985)	F133L and S257L	Melanoma and Colorectal	Rare (~1–5%)	B-RAF inhibitors	[1,11,12,15,18]
A-RAF (1986)	Rare Mutations	Lung, Ovarian, and Pancreatic	Rare	Limited therapeutic targeting due to complexity of RAF dimerization	
				Limited therapeutic options (less studied)	[1,11,15,16,18]
		Colorectal and Gliomas	Very Rare		[1,11,15,17,18]

**Table 2:** B-RAF inhibitors across cancer subtypes.

Cancer Type	B-RAF Mutation Prevalence	Primary Treatment	Mechanism of Action	References
Melanoma	~50% (V600E/K)	B-RAF inhibitors + MEK inhibitors (e.g., Trametinib, Dabrafenib, Vemurafenib)	Trametinib inhibits MEK1/MEK2, blocking MAPK signaling	[28,33,34,35]
Non-Small Cell Lung Cancer (NSCLC)	1–2% of adenocarcinomas	B-RAF inhibitors ± EGFR/ALK inhibitors	B-RAF inhibitors block MAPK pathway; EGFR/ALK inhibitors target alternative pathways	[36,37]
Hepatocellular Carcinoma (HCC)	Rare	Sorafenib (multi-kinase inhibitor)	Blocks RAF-1, B-RAF, VEGFR2, c-KIT, and PDGFR-β	[21,38]
Colorectal Cancer (CRC)	8–12% of metastatic cases	Combination therapy targeting the entire RAS–RAF–MEK–MAPK axis	Inhibition of B-RAF alone leads to CRAF and RAS-mediated reactivation of EGFR	[39]
Thyroid Carcinoma	40–45% in Papillary Thyroid Cancer	Sorafenib, Lenvatinib (RTK inhibitors)	Target RAF kinases and VEGF receptors	[38,40]

**Table 3:** B-RAF + MEK + AKT inhibitors.

Trial Number	Combination	Target Pathway	Findings	References
NCT01902173	Uprosertib + Dabrafenib + Trametinib	AKT + B-RAF + MEK	AKT inhibition reduces cell proliferation and survival. Concurrent Uprosertib + Trametinib lowered Dabrafenib exposure	[47]
NCT02974725	Naporafenib + Rineterkib or Trametinib	B-RAF + AKT + MEK	Partial response in NSCLC patients with B-RAF non-V600 and KRAS mutations	[48]

**Table 4:** B-RAF + MEK + CDK/mTOR inhibitors.

Trial Number	Combination	Target Pathway	Findings	Reference
NCT02871791	Exemestane + Everolimus + Palbociclib	CDK4/6 + mTOR	Studied CDK4/6 resistance in metastatic breast cancer; CBR was 18.8%	[49]

**Table 5:** B-RAF + MEK + HSP90 inhibitors.

Trial Number	Combination	Target Pathway	Findings	Reference
NCT02721459	Vemurafenib + Cobimetinib + XL888	B-RAF + MEK + HSP90	Good tumor response but high toxicity	[50]

**Table 6:** Novel Pan-RAF Inhibitors.

Trial Number	Combination	Target Pathway	Findings	Reference
NCT05355701	PF-07799933 ± Binimetinib	B-RAF + MEK	Inhibited MAPK reactivation in resistant B-RAF tumors	[51]

**Table 7:** Phase III clinical trials.

Trial Number	Combination	Target Pathway	Findings	Reference
NCT05566795	Tovorafenib vs. Chemotherapy	B-RAF	Phase III trial in pediatric gliomas with RAF alterations	[52]
NCT01909453	Encorafenib + Binimetinib vs. Vemurafenib	B-RAF + MEK	Longest follow-up data of B-RAF/MEK inhibitors in melanoma	[53]
NCT04657991	Encorafenib + Binimetinib + Pembrolizumab	B-RAF + MEK + PD-1	Phase III trial in cutaneous melanoma with 65% ORR	[54]
